# Study Protocol: effects of acupuncture on hot flushes in perimenopausal and postmenopausal women – a multicenter randomized clinical trial

**DOI:** 10.1186/1745-6215-9-70

**Published:** 2008-12-03

**Authors:** Kun-Hyung Kim, Kyung-Won Kang, Hee-Jung Jung, Ji-Eun Park, So-Young Jung, Jun-Yong Choi, Sun-Mi Choi

**Affiliations:** 1 Department of Medical Research, Korea Institute of Oriental Medicine, 483 Expo-ro, Yuseong-gu, Daejeon, 305-811, Korea

## Abstract

**Background:**

Hot flushes are the most frequent climacteric symptom and a major cause of suffering among menopausal women. The condition negatively influences many aspects of women's lives. To date, conventional hormone replacement therapy (HRT) is considered the most effective treatment for hot flushes. However, HRT is associated with a host of negative side effects. Complementary and alternative medical (CAM) approaches have been employed to relieve symptoms and to avoid these side effects. Acupuncture is one of the most strongly preferred CAM treatments for many diseases, causing few serious adverse effects, and is frequently used in Korea.

We aim to evaluate the effectiveness of Traditional Korean Acupuncture (TKA) in conjunction with usual care, compared to usual care alone, on hot flushes in perimenopausal and postmenopausal women in Korea.

**Methods:**

This study consists of a multi-center randomized controlled trial with 2 parallel arms. Participants included in the study will meet the following criteria: 1) a documented daily average hot flush score ≥ 10 for one week prior to the screening visit 2) not taking HRT and other pharmaceutical therapies which might affect hot flushes or other vasomotor symptoms.

While maintaining usual care, the treatment group will receive acupuncture 3 times a week, for a total of 12 sessions over 4 weeks. The control group will receive usual care alone during the same period. Post-treatment follow-up will be performed one month after completing 12 sessions of acupuncture.

**Discussion:**

This trial will provide evidence for the effectiveness of acupuncture as a treatment for hot flushes. The primary endpoint in both groups is a change in hot flush score from baseline to week 4 and/or week 8. As the secondary endpoint, we will employ the Menopause Rating Scale (MRS), a health-related quality of life questionnaire. Further analysis will examine the frequency, severity and difference in symptoms for daytime vs. nighttime hot flushes, sub-domain analysis of MRS, and participants' expectations of acupuncture treatment.

**Trial registration:**

Current Controlled Trials ISRCTN49335612

## Background

Hot flushes are the most frequent climacteric symptom and a major cause of suffering in menopausal women [1,2]. Diminished overall quality of life, social distress and emotional embarrassment are also frequent complaints due to menopause-associated hot flushes [3].

The prevalence of hot flushes varies according to ethnicity, region and cultural characteristics [4,5]. Most women experience hot flushes for 6 months to 2 years [4]. For many women, most menopause-related vasomotor symptoms including hot flushes tend to resolve spontaneously within a few months [6]. About 29% of 60-year-old women, however, report persistent hot flushes [6].

It is well known that the most effective treatment for hot flushes is hormone replacement therapy (HRT) [7]. Recent research performed by the Women's Health Initiative and the Million Women Study, however, suggests HRT may increase the risk of coronary heart disease [8], stroke [9], breast and endometrial cancer [10,11]. These findings regarding the adverse effects and potential risks of HRT have led to an expanded interest in non-hormonal therapies for managing hot flushes [12]. However, there are only limited data to support their use, and some adverse effects may be linked to certain types of non-hormonal therapies [13]. Herbs and phyto-estrogens have also been used in the clinic to alleviate menopausal symptoms, and many individual trials reported positive results for these therapies in reducing hot flushes. Such methodologies still require further research in terms of efficacy and long-term safety [14,15].

Acupuncture has been used worldwide to treat various conditions and diseases. In the context of clinical practice in Korea, acupuncture is one of the most frequently delivered modalities of medical treatment. In 1997, an NIH conference reached the consensus that acupuncture could be employed as a useful part of treatments in several clinical situations [16]. Since then, acupuncture has been increasingly adapted to treat a wide range of conditions and numerous related research efforts are currently being conducted around the globe. According to recent studies on the safety of acupuncture, it represents one of the safer forms of medical intervention with a low rate of adverse effects if performed by well-trained practitioners [17-19]. For treatment of hot flushes, acupuncture causes only minor side effects as opposed to conservative therapies, including HRT and non-hormonal agents, which cause more drastic adverse effects [8-11,13,17-19].

To date, acupuncture treatments have been increasingly utilized in several clinical trials for hot flushes due to menopause, breast cancer, and prostate cancer in males. Two randomized controlled trials (RCTs) (n = 24, n = 30) were carried out comparing electroacupuncture to sham acupuncture. These trials showed no significant differences between the two groups, but they did show considerable symptom reductions within each group [20,21]. One relatively large RCT (n = 103) showed no significant differences between real acupuncture and sham acupuncture [22], but a small RCT (n = 29) found that there was a significant difference between active acupuncture and placebo needles in terms of the severity, but not the frequency, of nocturnal hot flushes [23].

Notably, the first RCT (n = 52) using acupuncture for hot flushes in Korea was conducted with postmenopausal women. Comparing manual acupuncture to minimal acupuncture at the same points, the study showed no significant differences between the two groups, but also demonstrated significant symptom alleviation within both groups [24]. An unpublished study (n = 54) was carried out by the same group, comparing active acupuncture to placebo acupuncture, penetrating non-acupoints without reaching de-qi sensation on perimenopausal and postmenopausal women. The study failed to show significant differences between two groups, but showed a decrease in symptoms within groups. In breast cancer patients, one placebo-controlled RCT (n = 72) showed reduced hot flush frequency within the active acupuncture group, but no significant differences were found when comparing these subjects to Streightberger sham needle groups [25]. In prostate cancer patients that underwent castrational treatment, a small study (n = 31) employing two styles of acupuncture, electroacupuncture and traditional acupuncture, found that both treatment methodologies significantly lowered the number of hot flushes, as well as the distress from hot flushes (by 78% and 73%, respectively) [26].

As stated above, most research on the efficacy of acupuncture in the treatment of hot flushes failed to show any significant advantage of this technique as compared to the use of placebo needles. However, there were also some promising results which might support positive clinical implications; many studies found at least a 50% decrease of hot flushes within group after intervention. In one review regarding hot flushes, the authors reported a hot flush score reduction of greater than 50% by certain agents should be regarded as having greater effects than placebo effects [27]. Importantly, there is currently no satisfactory placebo acupuncture which is proven to be completely inert, so comparing acupuncture with placebo needles might underestimate the total effects of acupuncture [28]. In this study, we will investigate the comprehensive effects of acupuncture treatment in conjunction with usual care, as compared to usual care alone, avoiding the issues associated with placebo needling.

## Methods

### Inclusion criteria

Participants will be recruited from four centers (Seoul, Ilsan, Jecheon and Busan) by the use of local newspaper advertisements and posted notices at clinic cites. Participants include perimenopausal or postmenopausal women from 45 to 60 years old, who have average daily hot flush scores > 10 for one week prior to the screening visit (Hot flush scores: daily frequency × severity [0: none, 1: mild, 2: moderate, 3: severe, 4: very severe]). Serum TSH and free T4 levels will be tested to determine normal thyroid function.

We defined the term "perimenopausal" as menstrual irregularity or amenorrhea of 3–11 months [29,30], and the term "postmenopausal" as (1) 12 months of spontaneous amenorrhea or (2) 6 months of spontaneous amenorrhea with serum FSH levels > 40 mIU/ml or (3) 6 weeks postsurgical bilateral oophorectomy with or without hysterectomy or (4) hysterectomy with at least one intact ovary [31,32]. Despite the recommendation of the korpilampi workshop(1986) not to combine subjects undergoing natural menopause with those experiencing surgical menopause for analytic purposes [33], related studies which compare the risk of menopausal symptoms by the type of menopause are sparse and have shown conflicting results [34-36]. We thus included women undergoing menopause due to both natural causes and surgery. Instead, we will not only analyze data in a pooled population, but also perform a sub-analysis examining data separately for those with natural or surgical menopause.

### Exclusion criteria

Participants will be excluded when they are suffering serious medical conditions, such as uncontrolled hypertension, diabetes mellitus requiring insulin injection, any type of thyroid dysfunction, past or current malignant tumor, severe dyslipidemia, other infectious diseases or systemic diseases insufficient for acupuncture treatment.

Ineligible participants will also include those who used any hormones, antidepressants, gabapentin, selective serotonin reuptake inhibitors (SSRI) or sedatives. In the case of previous or current use of HRT, adequate wash-out periods will be dictated according to the type of HRT (i.e. oral administration, transdermal patch or injection).

Over the counter (OTC) drugs will be allowed for managing episodic colds, headaches and dyspepsia, etc. Some supplements including evening promise oil, phytoestrogens, omega-3 fatty acids, calcium and vitamins will also be permitted; however, participants will be gently advised to reduce the dose, stop taking the supplement, or at least refrain from adding another supplement to their regimen. Some OTC drugs containing black cohosh and human placenta extracts will not be allowed since there are concerns that those herbs might directly affect hot flushes and other vasomotor symptoms, in certain constitutions, according to Traditional Korean Medicine (TKM) theory. We will investigate the drugs taken by each participant at every visit and request that study participants notify us of any addition to their medication/supplement regimen. Any additional acupuncture treatment, herb prescription, or therapeutic intervention by another TKM doctor will not be allowed during the study. For the purpose of analyzing daytime and nighttime hot flushes separately, we will exclude participants who work at night. See eligibility criteria in table 1.

**Table 1:** Eligibility Criteria

Inclusion criteria
45–60 years old
Perimenopausal women (menstrual irregularity or amenorrhea of 3 – 11 months)
Postmenopausal women:
- 12 months of spontaneous amenorrhea
- 6 months of spontaneous amenorrhea with serum FSH levels > 40 mIU/ml
- 6 weeks postsurgical bilateral oophorectomy with or without hysterectomy
- hysterectomy with at least one intact ovary
Average daily hot flash scores > 10 for one week prior to screening visit

Exclusion criteria

Poorly controlled hypertension
Diabetes mellitus needed to be controlled by insulin injection
History of past or current malignant tumor
Any type of thyroid dysfunction
Severe dyslipidemia
Other infectious diseases or systemic diseases inadequate for acupuncture treatment
Other serious medical conditions
Use of HRT, antidepressants, gabapentin, SSRI's, sedatives
Use of unidentified herb prescriptions or herbs the investigator considers to affect hot flushes
Over-the-counter drugs which contain black cohosh or human placenta.
Working at night

### Interventions

TKA regimens designed according to TKM clinical experts will be used in this trial for alleviating hot flushes and vasomotor symptoms by balancing yin and yang in the participant. These regimens were also used in two previous studies [25], one of which is unpublished and mentioned above. The TKM doctors who conduct the treatments have practiced for at least 3 years in the clinic; they belong to each clinical center spread among different provinces in South Korea.

Participants will be randomly assigned to either the acupuncture treatment plus usual care group or the usual care alone group. The acupuncture treatment plus usual care group will receive acupuncture 3 times/week, for a total of 12 sessions. Six acupuncture points (ST36, SP6, LI4, PC6, HT7, HT8) will be inserted bilaterally in the four peripheral extremes and one point (CV4) will be inserted unilaterally in the lower abdominal region. TKM doctors will manually manipulate the acupuncture needles with de-qi sensation and maintain the needles for 20 minutes with intermittent manual stimulation. Infrared irradiation will be used to warm the lower abdomen during the 20 minutes of acupuncture treatment, as it is performed in conjunction with acupuncture, generally.

TKM doctors will use sterile, disposable needles with a length of 40 mm and a diameter of 0.25 mm (Dongbang Acupuncture Inc, Korea). They will be inserted to a depth of 3–15 mm, according to the points selected.

Participants who will be allocated to usual care alone group will receive no acupuncture treatments throughout the 4 weeks. They will record their hot flush symptoms in a daily diary. After four weeks, if participants elect to try the acupuncture treatment, the same treatment will be provided.

### Study outcome

The primary outcome measure is the hot flush score, produced by multiplying severity and frequency of hot flushes during a 24-hour period in a daily diary, which demonstrates validity and reliability in measuring hot flush activities [27]. Participants will be asked to record the number and severity of daily hot flushes during the entirety of the study period. Participants in both groups will also be routinely encouraged to fill out the diary by periodic short message service (SMS) and phone-call reminder both in order to avoid the selective loss to follow up, that is, selection bias and to prevent the indolence of writing daily diaries which are associated with participant knowledge-related bias in this unblinded trial. We will employ the slightly modified severity rating scale (0:none, 1:mild, 2:moderate, 3:severe, 4:very severe) suggested in the studies of Sloan et al. [27].

We will examine the reduction in hot flushes over a 24-hour period, as well as daytime and nighttime separately for sub-analytic purpose to check whether acupuncture has different effects on daytime versus nighttime hot flushes.

The secondary outcome measure is the Menopause Rating Scale (MRS) to assess other vasomotor symptoms. MRS is a highly qualified self-administered questionnaire for assessment of menopausal symptoms [37]. It contains 11 questions divided into 3 categories: psychological, somatic and urogenital.

We will also estimate the expectations of participants by using the credibility and expectancy questionnaire, aiming to determine whether expectations make a difference to outcome [38,39]. In order to assess expectations without potential influences by participants' knowledge of allocation results, a questionnaire will be administered to eligible women before notification of assignments. Data collection schedule is detailed in table 2.

**Table 2:** Data Collection Schedule

Measures	Screening	Baseline Randomization				End		Follow-up
Week number	-1	0	1	2	3	4	6	8

Sociodemographic characteristics	O							
Hot flush diary	O	O	O	O	O	O	O	O
Credibility and Expectancy questionnaire		O						
MRS		O		O		O	O	O
Medical history	O							
Physical activity	O							
Smoking/Alcohol	O							
Pharmaceutical therapies (OTC and prescribed drugs)	O	O	O	O	O	O		O
Herb medications	O	O	O	O	O	O		O
Adverse effects			O	O	O	O		
FSH, Estradiol	O							
TSH, Free T4	O							

In case of an unblinded trial, the rater(or assessor) knowledge of treatment assignment could modify the outcome assessment [40]. We employed only patient- reported and self-administered outcomes with concerns of potential rater bias in outcome assessment. However, related bias due to the unblindness might still occur despite all of efforts to prevent them. It should be recognized as an unavoidable limitation of this study design.

### Randomization

**Figure 1:**
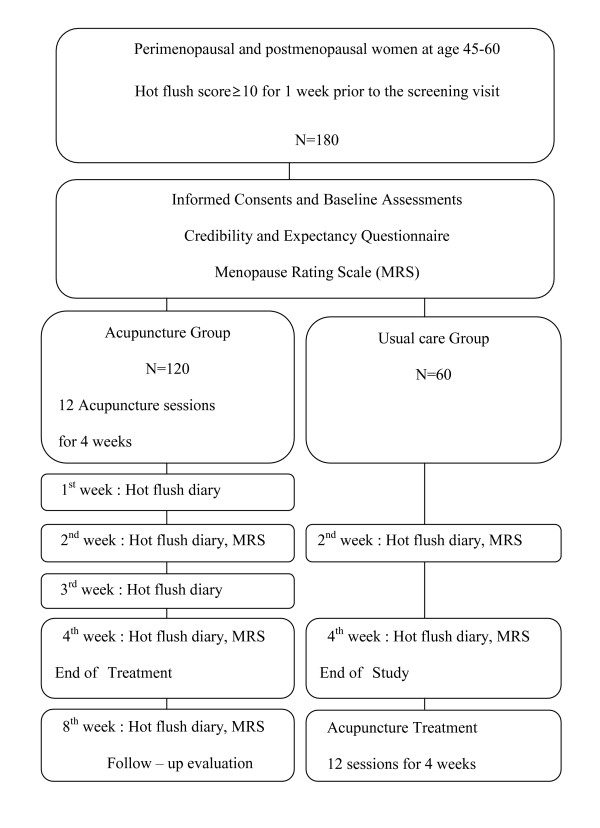
Flow diagram of study design.

Ultimately, 180 participants will be randomized to the acupuncture treatment plus usual care group or usual care alone group and divided into four centers (see Figure 1). Each center will recruit a total of 45 participants, with 30 assigned to the acupuncture plus usual care group and 15 to the usual care alone group. A block size of 3 is used to assign 2 for acupuncture treatment plus usual care group and 1 for usual care alone group. Separate randomization table will be provided for each center.

### Ethics

Oral and written consent will be obtained from each participant. This study was approved by the institutional review boards at the Dongguk International Hospital, East-West Neo Medical Center of Kyung Hee University, Semyung University Hospital and Dong Eui Medical Center.

### Statistical methods and sample size

Analyses will be performed for two populations: 1) an intention-to-treat population consisting of all randomized participants with at least one measurable outcome report following acupuncture treatment (missing data will be replaced with last observation values); 2) a per protocol population including only participants without major protocol deviations. All data will be analyzed descriptively. All main analyses will be based on the intention-to-treat population. For primary and secondary outcome measures, the mean differences from baseline values to the end of treatment will be compared using two-sample t-test or Wilcoxon rank sum test. A trend test will also be performed using repeated measures analysis of variance. We adjusted for possible confounders using criteria of statistical significance.

All adverse events reported during the study will be included in the charts; then incidences of adverse events will be calculated. The percentage of the subjects with adverse events in each group will be calculated and compared with the chi-squared test or Fisher's exact test value.

Statistical analyses will be performed using the SAS statistical package program (ver. 9.1.3) and the level of significance will be established at α < 0.05. In calculating the sample size, we assumed a power of 90% and α value of 5%, in order to detect a hot flush score difference of 15.34 with a standard deviation of 26.5 drawn by two previous studies in Korea [24,41]. Therefore the acupuncture plus usual care and usual care alone group (2:1 allocation) will include 120 and 60 participants, respectively, allowing for a 20% withdrawal rate. This implies a sample size of 45 for each center, with 30 participants per acupuncture treatment plus usual care group and 15 per usual care alone group.

### Data handling

Investigators will enter the information required by the protocol into the Case Report Forms (CRFs). Non-obvious errors or omissions are entered on data query forms, which will be returned to the investigational site for resolution.

The data from all centres are pooled and summarized with respect to demographic baseline characteristics, effectiveness and safety observations.

### Data and safety monitoring

Regular monitoring will be conducted for quality control. Also, investigators can be convened to discuss practical issues that may be encountered such as adjusting recruitment capacity within each center, dealing with serious adverse events, revising the protocol, as well as certain important issues that may be raised by investigators and participants.

The assessment of safety will be based mainly on the frequency of adverse events, which includes all serious adverse events. Information regarding adverse events will be summarized by presenting the number and percentage of participants experiencing any adverse event, with the information also divided by body system. Any other information collected (e.g. severity or relatedness to acupuncture treatments) will be listed as appropriate.

## Abbreviations

HRT: Hormone Replacement Therapy; CAM: Complementary and Alternative Medicine; TKA: Traditional Korean Acupuncture; MRS: Menopause Rating Scale; NIH: National Institutes of Health; RCT: Randomized Controlled Trial; TSH: Thyroid Stimulating Hormone; FSH: Follicle Stimulating Hormone; SSRI: Selective Serotonin Reuptake Inhibitor; OTC: Over The Counter; TKM: Traditional Korean Medicine; SMS: Short Message Service; CRF: Case Report Form.

## Competing interests

The authors declare that they have no competing interests.

## Authors' contributions

All authors participated in the conception and design of the trial. KKH drafted the protocol and wrote the final manuscript. KKW, as a biostatistician, conducted statistical design of the trial and wrote part of the statistical methods, data handling and monitoring sections. CSM is the principal investigator of this study. JHJ, JSY, PJE, JYC and CSM contributed to the research design and made critical revisions. All authors read and approved the final manuscript.
